# Treg Cell Therapeutic Strategies for Breast Cancer: Holistic to Local Aspects

**DOI:** 10.3390/cells13181526

**Published:** 2024-09-11

**Authors:** Hanwen Zhang, Oliver Felthaus, Andreas Eigenberger, Silvan Klein, Lukas Prantl

**Affiliations:** Department of Plastic, Hand and Reconstructive Surgery, University Hospital Regensburg, Franz-Josef-Strauss Allee 11, 93053 Regensburg, Germanylukas.prantl@klinik.uni-regensburg.de (L.P.)

**Keywords:** regulatory T cells, breast cancer, treatment

## Abstract

Regulatory T cells (Tregs) play a key role in maintaining immune homeostasis and preventing autoimmunity through their immunosuppressive function. There have been numerous reports confirming that high levels of Tregs in the tumor microenvironment (TME) are associated with a poor prognosis, highlighting their role in promoting an immunosuppressive environment. In breast cancer (BC), Tregs interact with cancer cells, ultimately leading to the suppression of immune surveillance and promoting tumor progression. This review discusses the dual role of Tregs in breast cancer, and explores the controversies and therapeutic potential associated with targeting these cells. Researchers are investigating various strategies to deplete or inhibit Tregs, such as immune checkpoint inhibitors, cytokine antagonists, and metabolic inhibition. However, the heterogeneity of Tregs and the variable precision of treatments pose significant challenges. Understanding the functional diversity of Tregs and the latest advances in targeted therapies is critical for the development of effective therapies. This review highlights the latest approaches to Tregs for BC treatment that both attenuate Treg-mediated immunosuppression in tumors and maintain immune tolerance, and advocates precise combination therapy strategies to optimize breast cancer outcomes.

## 1. Introduction

**Figure 1:**
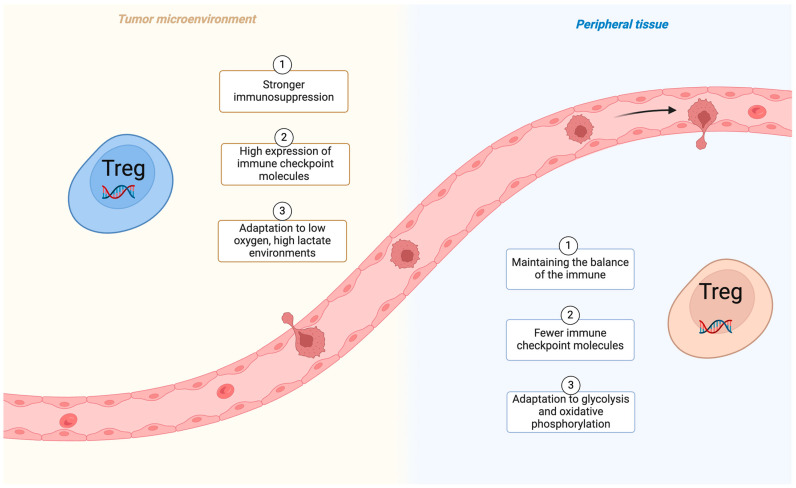
Tregs function differently in the tumor microenvironment and peripheral tissues (created with BioRender.com).

BC is the second most common tumor worldwide. The World Health Organization (WHO) counted 2.3 million women diagnosed with breast cancer globally in 2022 (11.6% of all cancer cases). Of these patients, 666,000 died (6.9% of all cancer deaths) [1,2]. Although good results have been achieved through surgical resection, radiotherapy and other means of treatment, the high variability of BC tumors and drug resistance increase the difficulty of treatment [3,4,5,6]. Currently the treatment of BC remains a major health challenge globally and requires new therapeutic strategies. It is well known that tumor-infiltrating lymphocytes (TILs) have emerged as important markers of healing and therapeutic relevance, and an increasing number of studies confirm the role of T cells in cancer immunotherapy [7,8,9]. Importantly, the ratio of CD4^+^ to CD8^+^ T cells correlates with tumor lymphatic metastasis and overall survival. The CD4^+^ T cell subclass, i.e., CD4^+^ CD25^+^ Foxp3^+^ Tregs, plays an important role in the tumor regulation of the immune response [10]. Numerous studies have shown that an increase in Tregs enhances tumor aggressiveness and reduces overall cancer survival [11,12,13]. The suppression of inflammation, modulation of autoimmunity, and control of tumor development and prognosis through Tregs have become popular areas of tumor immunotherapy in recent years [14,15,16]. This review explores the complex role of Tregs in BC progression and highlights recent advances in exploiting their tumor immunotherapy potential. Figure 1 demonstrates the status of different Tregs in tumor tissue and peripheral tissue.

## 2. Treg Classification and Characteristics

Tregs are a naturally occurring population of suppressor T cells located in secondary lymphoid organs and barrier tissues present in the body, such as the skin, lungs, gastrointestinal tract and liver [17,18]. When inflammatory conditions exist in the body, Tregs are recruited to the site of inflammatory injury and provide immunosuppression to reduce the immune response [19]. In addition to controlling autoimmunity, Tregs are able to inhibit antitumor immune responses and promote immune evasion and cancer progression [20].

### 2.1. Treg Classification

Tregs are classified according to the site of development: Tregs of thymic origin (tTreg) and Tregs of peripheral origin (pTreg) [20,21]. tTregs: CD4^+^ T cells in the thymus interact with each other via histocompatibility complex (MHC) class II-dependent T cell surface receptors (TCRs) in thymic stromal cells. The CD4^+^ CD25^+^ Foxp3^+^ Tregs that differentiate during natural maturation are active in the periphery. Their main role is the normal surveillance of self-antigens, as well as helping build up their own immune tolerance [22,23,24,25]. pTreg activates CD4^+^ CD25^−^ T cells in vitro via naive CD4^+^ T cells, generates Foxp3^+^ Tregs and suppresses effector T cells (Teff) through specific antigens and peptides [26,27,28].

**Figure 2:**
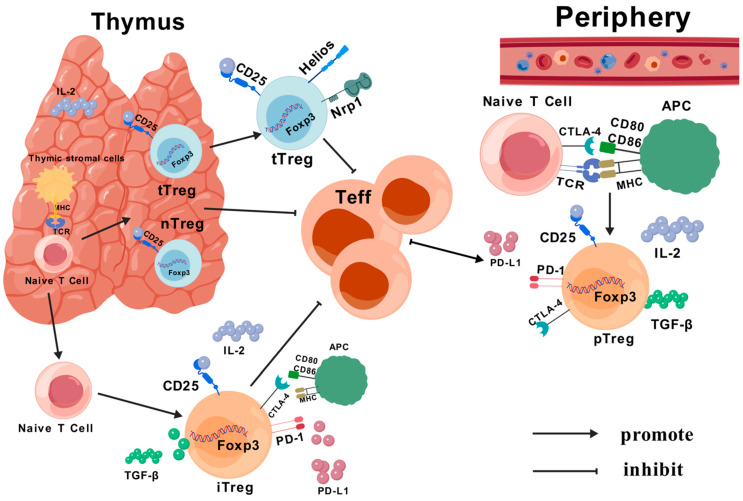
Source and classification of Treg cells: nTregs and tTregs secreted by naive CD4^+^ T cells in the thymus, and secreted in the periphery as iTregs and pTregs. tTregs produce an inhibitory effect on Teff, either through antigenic stimulation (IL-10, transforming growth factor (TGF-β), PD-L1, etc.) or through direct contact with CTLA4.

According to their biological properties, Tregs are divided into “natural” Tregs (nTregs) and “induced” Tregs (iTregs). nTregs are mainly CD4^+^ Tregs that mature after IL-2 and IL-15 stimulation in the thymus and maintain immune tolerance in peripheral areas of inflammation or tumor tissues, and account for approximately 1–3% of total CD4^+^ T lymphocytes [29,30]. In contrast, iTregs are also differentiated from naive CD4^+^ T cells after a series of antigenic stimuli. iTregs can be subdivided into various subtypes such as Tr1 cells (CD4^+^ CD25^−^ CD45RB^low^), Th3 cells (CD4^+^ CD25^low^) and CD8^+^ Treg (CD25^+^ Foxp3^+^) cells [31,32] (Figure 2).

Based on the degree of activation, Tregs can be further classified into central Tregs (cTregs) and effector Tregs (eTregs). cTregs are also known as naive Tregs and are mainly derived from peripheral and secondary lymphoid Tregs [33]. eTregs are found mainly in non-lymphoid organs, with a small fraction of them present in secondary lymphoid organs. Under pathological conditions, cTregs are converted to eTregs thereby releasing tissue repair molecules to repair tissues or suppress immune responses [34,35,36]. eTregs are antigenically activated in vivo, strongly express cytokines such as CD44, ICOS, CTLA-4, and PD-1, and play an important role in the suppression of Tregs [37,38].

**Figure 3:**
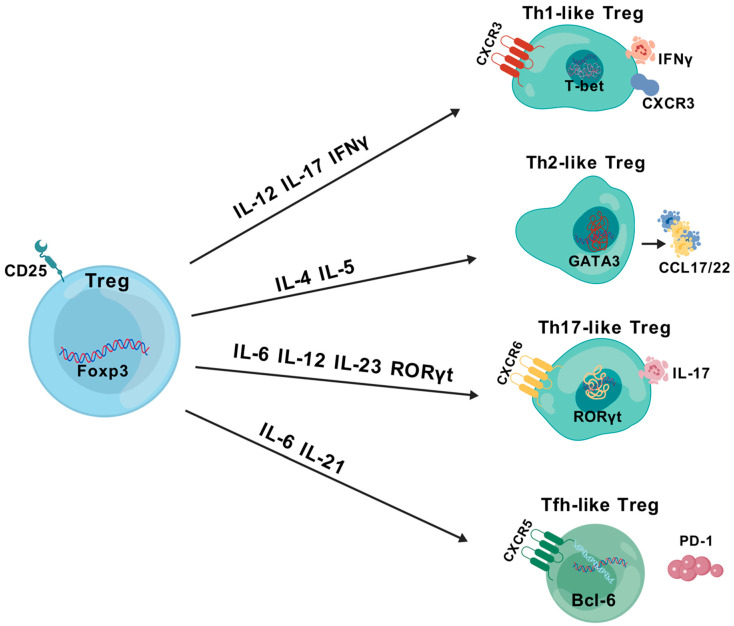
Plasticity of Tregs, in relation to Th cells in the immune system.

Classification according to appearance and function. Initially, CD4 T cells were classified into helper T cells (Th1, Th2, Th17) and iTregs. Later, classical CD4^+^ helper T cells were classified into a subpopulation of neo-memory Tregs, called Th-like Tregs [39,40] (Figure 3). Th1-like Tregs are characterized by the expression of T-bet and CXCR3, and CD4^+^Foxp3^+^ iTregs have been reported to produce the pro-inflammatory cytokine interferon γ (IFNγ) under the influence of the Th1 cytokine milieu, which rapidly develops and suppresses the initial immune response in Th1-like iTregs during the early stages of inflammation formation [41,42]. Th2-like Tregs significantly upregulate PTGDR2 (CRTh2) to prevent apoptosis, as well as the transcription factor GATA3, which regulates chemokines [43] and produces high levels of IL-4, IL-5, and IL-13 [38,44]. Th2-like Tregs also exhibit higher chemotaxis toward CCL17/22 than other Treg subpopulations, thereby increasing malignant migration [45,46]. Th17 and Tregs can be interconverted under specific conditions [47]. The differentiation of Th17-like Tregs is promoted primarily through a Th17-specific transcription factor, RORγt^+^, together with IL-2/IL6-associated STAT5 [48,49]. The accumulation of Th17 and Tregs in the tumor environment (TME) starts from the early stages of BC. In contrast, the increase in Treg infiltration and the conversion of Th1 cells to Tregs occurs in the middle-to-late stages of BC [50,51]. Follicular regulatory T (Tfr) cells are considered to be a special Treg population in the germinal center. They are induced by IL-6 and IL-21 after the stimulation of Tregs [52,53]. They retain their inhibitory function on the T cell receptor (TCR) as they retain factors such as the inducible T cell co-stimulatory factor (ICOS) and PD1. Tfr also expresses CXCR5, the transcriptional repressor B-cell lymphoma 6 (Bcl6), and secretes IL-10 directly or indirectly to regulate the immune function of B cells [54,55]. Overall, the plasticity of Tregs has characteristics associated with T helper cells, which increases the complexity of the tumor immune environment and suggests that Tregs are “double agents”.

### 2.2. Treg Characteristics

Tregs are characterized by the constitutive expression of the interleukin 2 (IL-2) receptor alpha chain (CD25) and transcription factor Foxp3 [56,57]. Foxp3 is an X-linked forkhead/wing-helix transcription factor characteristically expressed mainly by endogenous CD4^+^ CD25^+^ [23,58,59]. It forms complexes with IL-2 and IL-2R on the cell surface, and promotes Foxp3 expression through intracellular signaling after binding to CD25 (e.g., the Janus kinase (JAK)-STAT pathway, phosphatidylinositol 3-kinase (PI3K)-AKT pathway). Post-translational modifications of Foxp3 (e.g., methylation, acetylation and phosphorylation) can enhance or inhibit Treg function [12,60,61,62,63]. In BC, Foxp3 methylation modification is an important area of research. Methylation is catalyzed mainly by DNA methyltransferases (DNMTs), which add a methyl group to the 5′ position of cytosine to form 5-methylcytosine. This modification can inhibit gene expression [64,65]. Many studies have shown that Foxp3 CpG hypomethylation mediates Treg cell differentiation [64,66]. In BCs, high levels of methylation in the promoter region of Foxp3 stop its transcription, which lowers the amount of protein it makes [67]. Li J et al. found that BRCA patients with promoter hypomethylation had a good prognosis by examining Foxp3 expression in 123 BC samples and 5 BC cell lines [68]. An antitumor effect has been achieved by S-adenosylmethionine (SAM) to increase FOXP3 methylation to reduce the inhibitory capacity of Tregs [69]. The acetylation of Foxp3 occurs mainly at lysine-specific residues. Histone acetyltransferases (HATs), like p300 and CREB-binding protein (CBP), can add acetyl groups to certain parts of Foxp3. This makes Foxp3 more stable and improves its ability to carry out transcriptional functions [70,71]. The inhibition of Foxp3 acetylation at BC then started to become a potential therapeutic strategy for antitumor immune responses. It was suggested that inhibiting Foxp3 acetylation by using p300 inhibitors further reduced Treg activity and enhanced anti-breast cancer function in transgenic animals [72]. The phosphorylation of Foxp3 is usually regulated by a variety of protein kinases, such as protein kinase C (PKC), phosphatidylinositol-3-kinase (PI3K), Akt and MAPK [73,74]. Phosphorylation usually occurs on the serine or threonine residues of Foxp3, and this modification can affect the localization, stability and transcriptional activity of Foxp3 [75].

Similarly, Foxp3 can bind to the site-specific transcription factor STAT3. BC is induced to transcribe this factor via STAT3 in conjunction with the vascular endothelial growth factor A (VEGFA) promoter. VEGFA secreted by Treg promotes BC angiogenesis [76]. However, the role of Foxp3 in BC immunity remains controversial. A recent meta-analysis found that Foxp3^+^ TIL had better pathological complete remission (pCR) and overall survival (OS) in human epidermal growth factor receptor 2 (HER2^+^) BC and triple-negative BC populations [77]. Bioinformatics method analysis also found that Foxp3 expression was higher in breast-invasive carcinoma (BRCA) than in normal tissues. The overexpression of Foxp3 was associated with a better prognosis. Further studies found that significantly elevated Foxp3 mRNA levels were positively correlated with OS in BRCA patients [68,78]. Zuo T et al. found that the Foxp3 gene, a mammary tumor suppressor in mice and humans, interacts with the forkhead DNA-binding motif in the ErbB2 promoter to repress the transcription of the HER-2/ErbB2 gene [79]. However, the role of Foxp3 in BC is complex, suggesting its potential use as a diagnostic biomarker and immunomodulatory influence.

## 3. Identification of Tregs

### 3.1. Flow Cytometry

Flow cytometry (FC) is currently the most commonly used tool for the assessment of Tregs and their subtypes [80]. Previously, the expression of CD25 was used to identify Tregs on circulating CD4^+^ T cells, but only accounted for 1–2% of total peripheral blood CD4^+^ T cells [81]. Heterogeneity and instability in the characterization of CD25 on T cells were later found, with only 35% expressing CD4^+^Foxp3^+^CD25^high^ [82]. It was then suggested to use a Treg marker group consisting of CD3^+^, CD4^+^, CD25^+^, CD127^−^ and Foxp3^+^, which, in combination, allows for the identification of Tregs for sorting [83,84]. Plitas G. et al. found a higher percentage of Foxp3^+^ Tregs in BC tissue lymphocytes compared to lymphocytes in peripheral blood and normal tissues by using FC. They also suggested that CD25 expression could differentiate tumor-associated Tregs from non-Tregs [85].

Mass spectrometry flow cytometry (MC) is also known as CyTOF (cytometry by time-of-flight). It combines flow and mass spectrometry techniques using metal-labeled antibodies, allowing for the measurement of up to 50 markers in a single cell, while evaluating the complexity and heterogeneity of the immune response under study [86,87]. In the Hartmann et. al.’s study, CyTOF was used to metabolically remodel naive versus memory CD8^+^ T cells in vitro, and they identified metabolically suppressive cytotoxic T cells in human colorectal cancer [88]. MC is able to accurately distinguish between different subpopulations of Tregs. And its phenotypic characteristics and functional status in the BC microenvironment were analyzed in detail [89].

### 3.2. Transcriptomics

Single-cell RNA sequencing (scRNA-seq) is RNA sequencing performed at the level of the resolution of a single cell to detect the level of gene expression in the cell [90]. By analyzing the mRNA expression profiles of Tregs, it is possible to identify specific genes related to Treg function, such as FoxP3, CTLA-4, IL-10, and TGF-β [91]. scRNA-seq also allows for a detailed analysis of the heterogeneity of Treg cells in the breast cancer microenvironment [92].

### 3.3. Proteomics

Proteomics is the study of protein expression levels, post-translational modifications, protein–protein interactions, etc., from which information about the details of a disease is obtained at the protein level. In the treatment of BC, proteomics is able to identify specific functional proteins in Tregs, such as cytokines involved in immunosuppression, co-inhibitory molecules (e.g., PD-1, CTLA-4), and so on. The quantification of protein expression provides insight into the function of these proteins in BC and their regulatory mechanisms [93,94]. In recent years, high-dimensional radiomics have emerged as a novel biomarker discovery platform that can non-invasively measure Treg infiltration in BC patients at multiple time points, but obtaining high-quality data and large populations is currently quite difficult and requires further validation [95].

## 4. Treg Interactions in BC

In the beginning, BC was treated as a separate tumor, slowly evolving into a dynamic balance between a central tumor and a local surrounding microenvironment with complex systemic and local interactions. On the other hand, the TME is a bridge involved in the communication between the tumor and external substances [96]. Many studies have shown that the density of Tregs in BC is significantly correlated with localization, higher tumor grade, HER2^+^, ER negativity and a poor prognosis [97,98,99]. Expressing CD4^+^ CD25^+^ Foxp3^+^ Tregs exerts immunosuppressive functions under specific conditions in the TME, while tumor cells in the TME also regulate their own metabolism to adapt to the alterations in the TME, thereby promoting the differentiation of intra-tumorally infiltrating and the performance of their biological functions [100,101].

### 4.1. Chemokine Recruitment of Tregs

**Figure 4:**
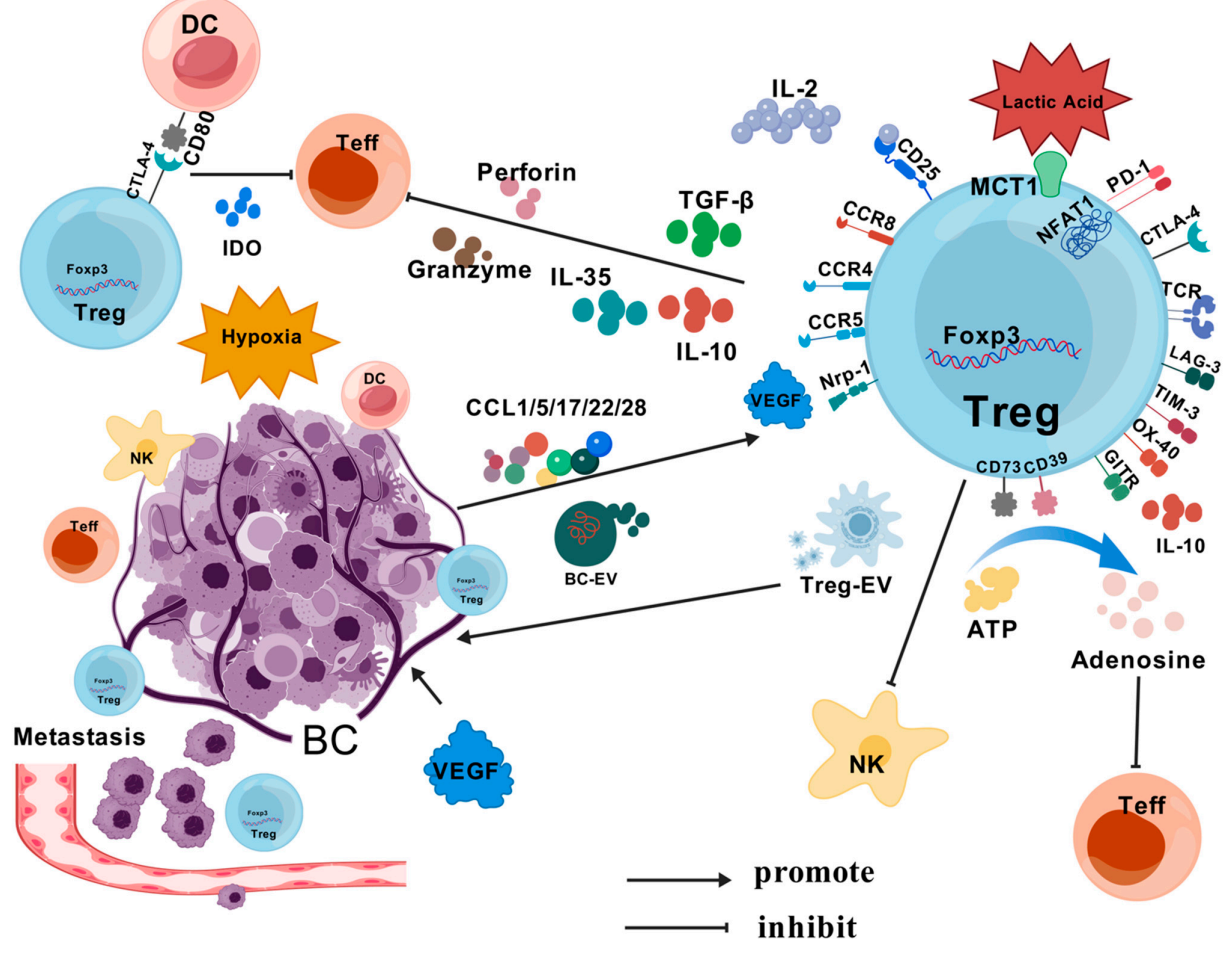
BC interacts with Tregs in the TME. Tumor tissue in the TME recruits Tregs via a variety of chemokines, leading to local Treg enrichment in the tumor (e.g., CCL17/22, CCL5, CCL1-CCR8, etc.). Tregs inhibit Teff through surface antibodies (CTLA-4, PD-1, etc.) and by secreting TGF-β and cytokines (IL-10, IL-35, etc.). Metabolites associated with Treg cells (ADO, IDO, lactate) can promote Treg activity, creating a positive feedback loop. Tregs also control NK activation in lymph nodes. VEGF attracts Foxp3^+^ Treg with high Nrp-1 expression to achieve a tumor immune escape. ASC-EV and Treg-EV promote each other.

Tregs are recruited by tumor tissues in the TME through a variety of chemokines, leading to Treg enrichment locally in the tumor (CCL17/22-CCR4, CCL5-CCR5, CCL1-CCR8, CCL28-CCR10 and CXCL9/10/11-CXCR3, etc.) (Figure 4) [102,103,104,105,106,107,108]. Many studies have demonstrated that some chemokines and receptors, such as CCR4-CCL17/22, CCR5-CCL5, etc., promote the metastasis of BC tumors to the lymphs and lungs, etc., mediated by Tregs [95,109,110]. Researchers are increasingly finding that activating or inhibiting chemokines either promotes or attenuates Treg infiltration, a therapeutic strategy that could potentially contribute to tumor immunotherapy.

### 4.2. Treg Immunosuppression of Tumors

Tregs promote tumor growth by inhibiting the role of tumor immune cells through a variety of means; a large body of evidence suggests that Tregs expressing CTLA-4 are responsible for the inhibitory effect on conventional T cells. Tregs suppress immune responses by affecting the APC activation of other T cells through CTLA-4 [111,112,113,114]. More importantly, CTLA-4 binds to APC more strongly than CD28 and B7, disrupts APC maturation and proximally suppresses immunity after generating inducible inhibitory signals [115,116]. PD-1 on the surface of Tregs is thought to inhibit the function of effector T lymphocytes in non-lymphoid organs. The formation of the PD-1/PD-L1 axis inhibits T cell activation signaling via the T cell receptor (TCR), which can enhance Treg function and produce additional effector T cell suppression [117,118,119]. In addition to this, Tregs can also directly inhibit Teff by secreting TGF-β and cytokines such as interleukin-10 (IL-10) and interleukin-35 (IL-35) secreted by granzymes and perforins. They also inhibit dendritic cell (DC) function through the induction of the Smad signaling pathway and activation of STAT3 by TGF-β and IL-10 [120,121,122]. Glucocorticoid-induced tumor necrosis factor receptor (TNFR)-associated protein (GITR) also increases Treg expansion and promotes IL-10 production [123]. The infiltration and interaction of Tregs and BC are presented in Figure 4.

### 4.3. BC Metabolism and Treg Effects

The extracellular and pericellular accumulation of adenosine (ADO) directly upregulates Tregs by regulating CD39 and CD73, and also through the type 1 purinergic adenosine receptor inhibiting the effector function of activated T cells [124,125,126,127]. Many studies have demonstrated the association of indoleamine 2,3-dioxygenase (IDO) with tumor-induced immunosuppression. The expansion of Tregs was induced by IDO in myeloid-derived suppressor cells (MDSC) [128,129]. Increased IDO expression in BC allows for Tregs to synergistically mediate lymphatic metastasis (Figure 4). It also exerts its inhibitory effects by reducing the local production of tryptophan and cytotoxic metabolites in the lymphatic drainage zone of tumors [130,131].

Tregs are increasingly adapting and promoting TME stabilization in low-glucose, high-lactate, hypoxic TMEs [132,133]. Kumagai et al. demonstrated that Tregs in high-lactate environments actively uptake lactic acid (LA) via the monocarboxylate transporter protein 1 (MCT1), which promotes the translocation of NFAT1 to the nucleus and enhances PD-1 expression [134]. CD36 also regulates mitochondrial adaptation through peroxisome proliferators, thereby increasing the activation of receptor-β signaling, further adapting to the lactate environment in the TME by programming Tregs [135]. In highly glycolytic triple-negative BC (TNBC), rapid glucose depletion leads to the accumulation of lactic acid, which can drive initial T-cell polarization toward Tregs. It also favors Treg survival and phenotypic integrity [132,136]. Tregs can also adapt to the unusual TME by increasing Glut1 expression and thus glycolysis rates [137].

### 4.4. Tregs Enhance Tumor Progression, Metastasis and Drug Resistance

In addition to suppressing tumor immunity, Tregs can directly influence the properties of tumor stem cells (CSCs). Xu et al. studied Tregs from mice that had mammary tumor Foxp3-EGFP and found that Tregs in the TME could support the mammary CSC phenotype through a paracrine effect. This led to an increased expression of stemness genes like Sox2 in BC cells, which improved their ability to form spheres and even made the tumor resistant to drugs [138]. In a recent study, Tregs were also found to mediate the CCL5/CCR5 promotion of BC axillary lymph node metastasis in peripheral blood and tumor tissue cells from BC patients analyzed by flow cytometry [95]. In a mouse BC model, Tregs also controlled the activation of natural killer (NK) cells in lymph nodes to promote BC lymph node metastasis [139], as an increased function of CCL28 in the hypoxic environment within the TME can promote Treg recruitment and increase the level of tumor-derived VEGF secretion [140]. At the same time, VEGF also attracts Foxp3^+^ Tregs with a high expression of Nrp-1 to help tumors achieve immune escape [141].

Adipose stem cells (ASCs) can be secreted from the large amount of adipose in breast tissue. On the other hand, the extracellular vesicles (EV) secreted by ASC (ASC-EV) can promote the differentiation of CD4^+^ T cells to Tregs and the metastasis of MCF-7 cells [142]. EV secretion from adipose mesenchymal stem cells (MSCs) has also been shown to promote Th17 and Treg responses with miR-10a, while reducing Th1 responses [143]. Also, the EV that comes from BCs (BC-EV) carries the lncRNA SNHG16, raises the expression of CD73 and changes T-cells into Tregs when miR-16-5p acts as competitive endogenous RNA (ceRNA) [144]. Interestingly, Tregs can also release exosomally characterized “disc-shaped” lipid bilayer vesicles (Treg-EV), which simultaneously exhibit the inhibition of CD4^+^ T cell proliferation (Figure 4) [145]. Numerous studies have found Treg-EV to play an important role in Treg-mediated immunosuppression, but the treatment of BC has received relatively little research. However, the relationship between Tregs and tumors or autocrine exosomes shows great promise [146,147,148].

Overall, Tregs and BC cells promote tumor progression and metastasis through various signals, molecules, and other interactions, and it is the plasticity of Tregs with detrimental effects on the antitumor that provides opportunities for therapeutic intervention in tumors.

## 5. Immune-Targeted Therapy against Tregs

### 5.1. CTLA-4-Targeting Antibody

**Figure 5:**
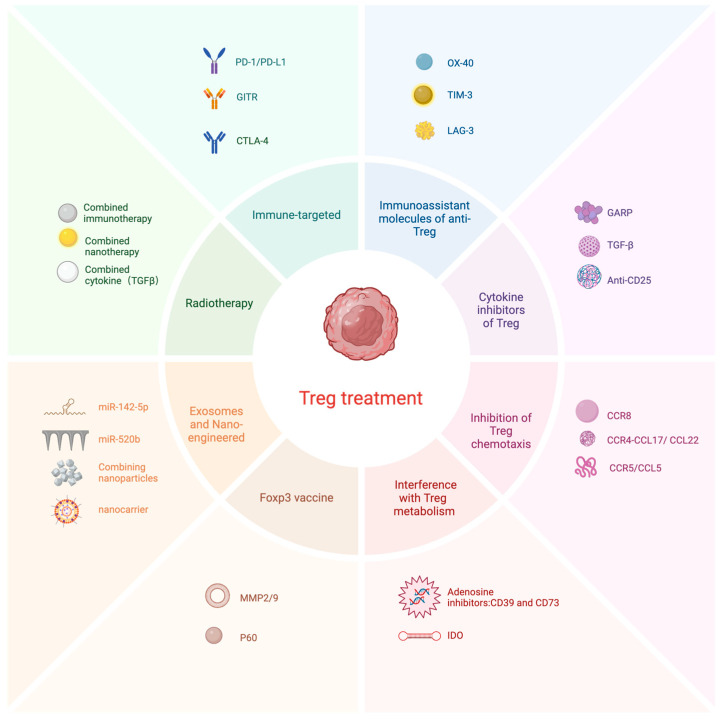
Treg removal or suppression methods in BC (created with BioRender.com).

CTLA-4 is an immune checkpoint molecule highly expressed in Tregs that inhibits the function of antitumor Teff while preventing the inappropriate and prolonged activation of Teff [149]. Ipilimumab was the first anti-CTLA-4 antibody approved for use in 2011 and was applied to unresectable melanoma patients with an overall remission rate of approximately 10% [150,151]. In BC, CTLA-4 is a co-suppressor receptor molecule expressed on activated T cells and Tregs that interacts with the B7-1 (CD80)/B7-2 (CD86) ligand-binding site on antigen-presenting cells (APCs), and competes with CD28 to negatively regulate T cells [152,153]. The blockade of FcγRIIB was found to restore the Treg-depleting ability and antitumor activity of Ipilimumab in a mouse model treated with CTLA-4 and Fcγ receptor (FcγR) humanization [154]. Ipilimumab induces immune-related adverse events (irAEs), resulting in patients not being able to benefit from long-term treatment. Thus, Gan X et al. developed a fully humanized pure heavy-chain antibody (HCAb 4003-2) against CTLA-4 that showed enhanced anti-Treg ability, reduced blood levels and irAE occurrence. This treatment provided more potent antitumor activity than Ipilimumab, with an improved safety profile [155]. An overview of common immune targets and other treatment options for Treg can be viewed in Figure 5.

### 5.2. PD-1/PD-L1-Targeted Antibodies

PD-1 belongs to the CD28 family and is a transmembrane protein consisting of 268 amino acid residues. PD-1 has two ligands: PD-L1 (B7-H1) and PD-L2 (B7-DC). PD-1/PD-L1 is highly expressed on the surface of APCs such as B cells and DCs, and on the surface of many malignant tumor cells. The main function of anti-PD-1/PD-L1 antibodies is to reverse T cell depletion [156]. Researchers have found that combining PD-1/PD-L1 inhibitors with neoadjuvant chemotherapy increases the rate of pathologically complete remission in early BC [157]. In a recent study in a mouse model of 4T1 BC, a combination of anti-PD-1 antibody and DBDx (dipyridamole, betadine and dexamethasone) was found to result in lower Treg ratios in peripheral blood and tumors, and higher CD8^+^ T/Treg ratios after treatment [158]. Since PI3Kδ inhibitor (YY20394) eliminated the downstream biological effects on Treg proliferation and function, YY20394 was combined with anti-PD-1 in mouse experiments, sensitivity to immune-blocking therapy was restored by inhibiting Treg function and synergistic tumor inhibitory effects demonstrated [159]. To address PD-1 blockade resistance in TNBC, Fattori S et al. used an Fc-optimized IL2+ anti-CD25 antibody in combination with anti-PD-1. The results increased the Teff/Treg ratio in tumors and promoted systemic antitumor immunity [160].

### 5.3. Anti-GITR-Targeting Antibodies

GITR (TNFRSF18/CD357/AITR) is highly expressed on the cell surface of Treg but is lowly expressed in naive T cells [161]. The modulation of the signaling axis between GITR and its ligand (GITRL) has been shown to inhibit Treg function and upregulate Teff cells in animal tumor models [162,163,164]. Additionally, through the anti-GITR agonist antibody DTA-1 mAb, it is possible to modulate the DTA-1-GITR axis to reduce Treg activity and quantity and thereby suppress tumors [161,163]. Researchers initiated the first human phase I trial of GITR agonism using the anti-GITR antibody TRX518, which led to a decrease in circulating and intra-tumoral T reg cells and an increase in the Teff/Treg ratio [165]. To treat larger tumors forming in the clinic, an increasing number of scholars are combining different types of therapies. In BC models, the combination of cisplatin or paclitaxel with anti-PD-1 and anti-GITR had a significant effect in 80% of mice within 90 days. The use of anti-GITR antibodies and anti-CTLA4 antibodies in MethA and CT26 tumor models resulted in increased levels of Teff and IFNγ [166,167]. A recent study found that the combination of local radiotherapy (RT) with DTA-1 mAb significantly enhanced the antitumor effect of anti-PD-L1 antibody in a TNBC model inoculated in 4T1 mice. It resulted in an increase in CD8^+^ T cells and a decrease in Tregs [168].

### 5.4. Immunoassistant Molecules of Anti-Treg

Lymphocyte activation gene-3 (LAG-3), T-cell immunoglobulin and mucin-containing protein-3 (TIM-3) and OX-40 are less reported immunoadjuvant molecules, but they are equally involved in the regulation of tumor cell immunity [169,170,171]. LAG-3 is a molecule that inhibits T-cell proliferation and activation. It is co-regulated by TILs, depleted T-cells and Tregs. However, its transmission pathway remains unclear [169,172]. According to Asano Y et al., immunohistochemistry in 177 patients with resectable early BC found that LAG-3 may be a good independent predictor of highly malignant BC, such as TNBC and HER2^+^ BC [173]. Combining anti-LAG-3 antibodies with paclitaxel in phase I and II clinical studies in BC increased the efficiency from 25% to 50% by co-suppressing Tregs [174]. Wildiers H et al. recently added a soluble dimeric recombinant form of LAG-3 in a phase IIb clinical study. The study treated 114 patients with metastatic BC Eftilagimod-α (IMP321) in combination with Zitotriol, and showed that LAG-3 did not significantly improve overall survival, but demonstrated the safety of LAG-3 and the palliative of tumors, and is a promising new strategy [175,176].

TIM-3 not only acts as an immune checkpoint modulator and synergistically inhibits T cell function with PD-1, but also suppresses the natural immune response of myeloid cells and plays an important role in cancer cell support and survival [170]. TIM-3 does not depend on the immunoreceptor tyrosine inhibitory motifs of the T-cell antigen receptor signaling regulatory locus, and so, TIM-3-based immune targeting may mitigate normal tissue damage and may ultimately lead to accurate and effective therapy [173]. In a nude mouse model study of adenocarcinoma cells, the combination of anti-PD-1 antibody and anti-TIM-3 increased human leukocyte antigen (HLA)-A2-restricted melanoma-associated antigen A11 (MAGE-A11) peptide. This resulted in the activation of induced cytotoxic T lymphocytes (CTL) and inhibited BC development [177].

OX-40 is expressed by activated T cells and is a member of the TNFR family, originally found to be expressed in rats with autoimmune encephalomyelitis. It was later demonstrated that the involvement of OX-40R during tumor initiation in vivo may enhance the function, expansion and survival of tumor-specific CD4^+^ T cells [178,179]. The sequencing of OX-40 by RNA-Seq in liver and colon cancer tissues revealed that it was overexpressed on tumor-infiltrating CD4^+^ T cells. The use of Fc-engineered αOX40 antibody (αOX40_v12), which selectively enhances affinity for FcγRIIB, resulted in the amplification of peripheral TILs [180]. OX40 participates in CD4^+^ T cell processes and opens new avenues for tumor immune-targeted therapies, despite the need for further refinement and discovery in OX-40 studies.

### 5.5. Cytokine Inhibitors of Treg

#### 5.5.1. Anti-CD25 Antibody

Il-2 is an important cytokine involved in Treg survival and function, and CD25 (IL-2Rα chain) is an indispensable growth factor for Treg development and homeostasis. Many experiments have found that injecting anti-CD25 antibodies decreases the number of peripheral CD4^+^CD25^+^ Tregs [181,182,183]. Anti-CD25 monoclonal antibodies (e.g., daclizumab, baliximab, etc.) have been shown to target CD25^+^ Tregs, thereby enhancing tumor immunity and immunotherapy [184,185], and daclizumab also significantly eliminates CD4^+^CD25^+^ Tregs from peripheral blood within 3–4 weeks and restores the Treg phenotype within 8 weeks [186]. Teff development and function will be impaired because the non-selective anti-CD25 antibody blocks IL-2 signaling required by T cells [187,188]. To ensure the targeted depletion of tumor-associated Tregs while avoiding immune-related side effects due to systemic Treg cytopenia, a specific selective CD25 blocker (RG6292) has been developed which enhances antitumor activity without significant immune-related toxicity [189]. Similarly, a CD25-based pyrrolobenzodiazepine (PBD) dimeric antibody–drug coupling (ADC) depletes Tregs in mice bearing MC38 tumors, while CD8^+^ Teff cells are unaffected [190]. There are also chimeric antigen receptor (CAR)-based NK cells engineered to express CD25 CAR constructs that can target CD25 Tregs, a novel CAR that provides new ideas for immune anticancer therapy [191].

#### 5.5.2. TGF-β and GARP

TGF-β, a key factor in tumor immunosuppression, can directly promote the proliferation of Treg cells. It can also achieve tumor immunity by inhibiting Teff cell effects, as well as DC survival and function [192,193]. Lainé A et al. administered anti-Itgβ8 antibody to β8-chained Tregs expressing the αvβ8 integrin (Itgβ8) in a mouse model of mammary cancer and melanoma. This treatment was found to inhibit TGF-β signaling and enhance the cytotoxic function of T cells [194]. By inhibiting TGF-β family members, activin A and TGF-β can effectively downregulate the increase in Tregs after radiotherapy, thus enhancing the effect of radiotherapy and effectively suppressing tumors [195,196]. It has also been found that in tumor fibroblasts, TGF-β was associated with resistance to anti-PD-1 antibodies. Once combined, anti-TGF-β and anti-PD-1 treatment can promote Teff cell infiltration and enhance antitumor capacity [197]. A recent study identified a bispecific antibody called AxF (scFv) 2 (which doubly blocks PD-L1 and TFG-β). When combined with tri-specific T-cell inducer (TriTE) or CAR-T, it significantly enhanced T-cell activation and improved survival in breast, lung and colorectal cancer models [198]. Currently, anti-TGF-β anti-immunity still lacks specificity, so it has not been incorporated into clinical applications for the time being. However, a growing number of studies have shown that combination therapy with anti-TGF-β and anti-PD-1 can be more effective than monotherapy, and that such regimens can be effective in reducing drug resistance while synergistically facilitating Teff’s fight against tumors.

Glycoprotein A repetitions predominant (GARP) is a transmembrane protein and is highly expressed in BC, and increased levels of TGF-β when GARP is overexpressed have been found to be tumor-inducing in mouse mammary tissue model experiments [199,200]. In contrast, lentivirus-mediated downregulation of GARP expression leads to the downregulation of Foxp3 and reduced Treg suppression [201]. GARP is also closely related to TGF-β and its function as a potential TGF-β receptor [202]. Therefore, numerous studies have shown that blocking the GARP–TGFβ1 complex is a promising option. Powderly J et al. used a monoclonal antibody called ABBV-151 to block the binding of the GARP–TGFβ1 complex. This antibody specifically interferes with the release of TGFβ1. Combining ABBV-151 with an anti-PD-1 antibody resulted in a stronger antitumor effect [203]. Satoh K et al. found that anti-GARP-TGF-β1 antibodies do not require FcγR-dependent function to exert antitumor activity in mice, as they do not lead to the depletion of Tregs causing subsequent autoimmune adverse reactions [204]. A recent study found that a novel naphtholactam platinum anticancer drug (NPt) can be stored in Treg lysosomes via ATP-binding cassette subfamily B member 9 (ABCB9) and tumor necrosis factor receptor-related factor 3-interacting protein 3 (TRAF3IP3). It specifically inhibits the activation of the GARP/TGF-β1 complex [205].

### 5.6. Inhibition of Treg Chemotaxis

#### 5.6.1. CCR8 Inhibitors

CCR8 is a cell surface receptor belonging to class A of the G protein-coupled receptor (GPCR) family and is considered one of the best tumor Treg targets [206,207]. In recent years, the prospect of Treg-mediated cancer immunotherapy against CCR8 has received widespread attention. Screening by multi-omics analysis with the Cancer Genome Atlas (TCGA) revealed that CCR8 is preferentially expressed on Tregs in BC [208,209]. The anti-CCR8 antibody of rat IgG2b isotype was injected intravenously into EMT6 BC cell-treated mice and was found to highly inhibit tumor growth with greater than 50% tumor clearance. Surprisingly, their findings also revealed that CCR8^+^ Treg depletion was not accompanied by harmful autoimmunity [109]. To increase the efficiency of such approaches, CCR8^+^ Tregs can also be selectively eliminated by Fc-optimized non-fucosylated (nf) anti-human CCR8 antibodies, which achieve dose-dependent, long-term effective antitumor immunity and synergize with PD-1 blockade [210]. Weaver JD et al. used a new Fc-optimized CCR8 antibody (GS-1811) to get rid of cells that only expressed CCR8 in a mouse tumor model. This antibody worked well with PD-1 inhibition to boost antitumor responses in a PD-1 resistance model [211]. A highly selective CCR8 antagonist (IPG7236) was the first antibody to enter this class of clinical trial phase, which exhibited anticancer effects through downregulation of Tregs and increase of CD8^+^ T cells. In a human BC mouse xenograft model, IPG7236 significantly inhibited tumour growth alone or in combination with a PD-1 antibody [212]. CCL-1, as a receptor for CCR8, has been shown to play an important role in Treg developmental transformation and Treg recruitment in BC. Inhibition of Treg function using a CCL-1 inhibitor (α-CCL1) can achieve antitumor effects [104,213].

#### 5.6.2. CCR4-CCL17/CCL22 Antagonists

CCR4 is a chemokine receptor for the metastatic process of Tregs and in tumor-infiltrating Foxp3^+^ Treg populations, most of which express CCR4 [214]. Thus, anti-CCR4 antibodies are increasingly being used as an adjunct to antitumor therapy [215,216]. Clinical trials of a humanized CCR4 antibody (Mogamulizumab) have focused on the treatment of advanced tumors such as T-cell lymphoma and leukemia [217,218]. A significant reduction in BC lung metastases was found by injecting anti-CCR4 antibody (TARC-PE38) in mice inoculated with 4T1 BC cells [110]. The oral CCR4 antagonist FLX475 was shown to be well tolerated to maximizes the suppression of Treg levels. Similarly, FLX475 monotherapy and combination with pembrolizumab for advanced tumors are undergoing clinical trials (NCT03674567, NCT03674567) [219]. In BC lymphocyte recruitment, CCL17/CCL22 acts as a ligand for CCR4, and CCL17/CCL22 is closely associated with Foxp3^+^ tumor-infiltrating regulatory T cells (Ti-Tregs) [220]. Marshall, L.A. et al. added a selective small molecule CCR4 antagonist (CCR4-351) to a mouse model of BC in vivo and in vitro, which blocked CCL17 and CCL22, ultimately leading to Treg suppression [221]. Although CCR4 has pro-growth and metastatic effects on BC, there are fewer experimental results on the efficacy of anti-CCR4 antibodies in BC, and further validation and exploration are needed.

#### 5.6.3. CCR5/CCL5

The chemokine CC motif-coordinated-5 (CCL5), also known as activation-regulated, and normally expressed and secreted by T cells, is a cell membrane protein that is a member of the G protein-coupled receptor superfamily [222]. It has been shown that CCL5 recruits Tregs via CCR5 and stimulates TGF-β to block the tumor killing function of CD8^+^ T cells [223]. Maraviroc (MVC) was originally used as a therapeutic HIV antiretroviral CCR5 blocker, and as one of the most valuable blockers in the anti-CCL5-CCR5 axis, it is widely used. Pervaiz et al. has demonstrated that blocking CCR5 with MVC reduces the proliferation and migration of metastatic breast cancer, and the drug significantly inhibited BC (MDA-MB-231) bone metastasis in nude mice [224]. MVC also prevents monocyte recruitment to tumors and significantly inhibits tumor growth in the rapidly progressing tumors (PT) of periductal mesenchymal cells in the breast [225].

### 5.7. Interference with Treg Metabolism

#### 5.7.1. Adenosine Inhibitors

Many studies have shown that Tregs are highly expressive of the extracellular nucleic acid exonucleases CD39 and CD73. They produce adenosine via intra-tumoral ATP and play a role in tumor immunosuppression. Therefore more and more studies are finding that adenosine signaling may be a key target for cancer therapy [226,227,228,229]. Extracellular adenosine is known to activate cellular signaling pathways via one of four G protein-coupled adenosine receptors: A1, A2A, A2B and A3. The proliferation of adenosine in the pericellular region has been shown to upregulate both the activity and amount of Treg, and to inhibit Teff inhibition via stimulation of the type 2 purinergic adenosine receptor (A2AR) [230,231,232]. In contrast to the manifestation of significant tumor resistance found in mouse models knocked down for CD39 and CD73, tumor rejection was also seen in A2AR-deficient animal experiments [233,234,235]. A recent study collected transcriptomic and pathological data from 1904 BC tumors (124 fresh biopsies) by PCR. The results showed that A2AR protein was positively correlated with PD-1 protein and negatively correlated with CTLA-4 protein, and was involved in tumor proliferation and metastasis [236]. SRF617, a novel anti-CD39 antibody, regulates CD39 in vivo immune cell levels and allows for an increased infiltration of CD8^+^ T cells in the TME [237]. Menzel S et al. used a 15 kD monomeric nanoscale antibody (SB24) that effectively antagonizes soluble CD39 and the ATP degradation of cell surface CD39 [238].

The use of anti-CD73 antibody inhibited tumor growth in E0771 BC mice and also improved the outcome of tumor radiotherapy [239,240]. Researchers have found that in preclinical models of solid tumors such as breast and colorectal cancer, the combination of an anti-A2AR antibody (CPI-444) with an anti-PD-1 or anti-CTLA-4 antibody inhibits tumor growth and is more effective than monotherapy [237]. In order to improve the effectiveness of anti-A2AR antibodies, more and more studies have begun to use them in combination with other drugs, greatly increasing the direction of application.

#### 5.7.2. Indoleamine 2,3-Dioxygenase (IDO) Inhibitors

IDO is an important player in the regulation of T cell proliferation and metabolism in cancer. Indoleamine 2,3-dioxygenase 1 (IDO1) is a tryptophan catabolic arthritase that is abundantly expressed in various metastatic tumors [241]. Clinical trials of indolimod (1-D-MT) (NCT01042535) in combination with adenovirus-p53, epalrestat (NCT03291054), and nafpurimod (GDC-0919) (NCT02471846) in combination with atezolizumab have been conducted in BC, but the results are subject to further study [242]. Combination therapies containing anti-CTLA-4, anti-PD-1/PD-L1 and IDO1 inhibitors have been shown to significantly downregulate Treg levels in vivo [243]. Studies of CSCs in a 4T1 BC tumor model also revealed that by inducing immunogenic cell death (ICD) and blocking IDO, Treg numbers and tryptophan depletion can be reduced and CSCs effectively eliminated [244]. These preliminary studies are exciting, but further studies are needed to elucidate more valuable clues about IDO inhibitors on cancer.

### 5.8. Foxp3 Vaccine

The importance of the transcription factor Foxp3 for Tregs and the uncertainty about the prognosis of BC have already been mentioned. However, vaccines against Foxp3 are still considered a way to deplete Tregs, since Foxp3 is a nuclear product and is not expressed on the membrane surface of Tregs. Therefore, the use of monoclonal antibodies is unable to act on Foxp3, and Foxp3 mRNA-transfected DCs are beginning to be used as vaccines. Inoculation into melanoma and thymoma mice preferentially depleted Foxp3^+^ Tregs, and the cytotoxicity of the vaccine was comparable to that of anti-CD25 antibodies [245]. Candia et al. started to use a binding peptide (P60) that affects Foxp3 nuclear translocation and could effectively inhibit the tumor-intrinsic effect of Foxp3 in experimental BC, and reduces tumor cell viability and migration [246]. Ding et al. combined P60 with the tumor tissue-specific matrix metalloproteinase protease 2/9 (MMP2/9) to form fusion proteins 6 (P60-MMPs), which can be specifically targeted to the interior of 4T1 BCs for degradation to P60. Once P60 binds to Foxp3, it downregulates the activity of Tregs and inhibits BC cell growth and lung metastasis [247]. Future research should continue to delve deeply into the complex relationship between Foxp3 and breast cancer. Researchers should delve deeper into understanding the connection between Treg cells and cancer by examining their interactions with other proteins, the control of gene expression and their impact on the immune system.

### 5.9. Exosomes and Tregs

**Figure 6:**
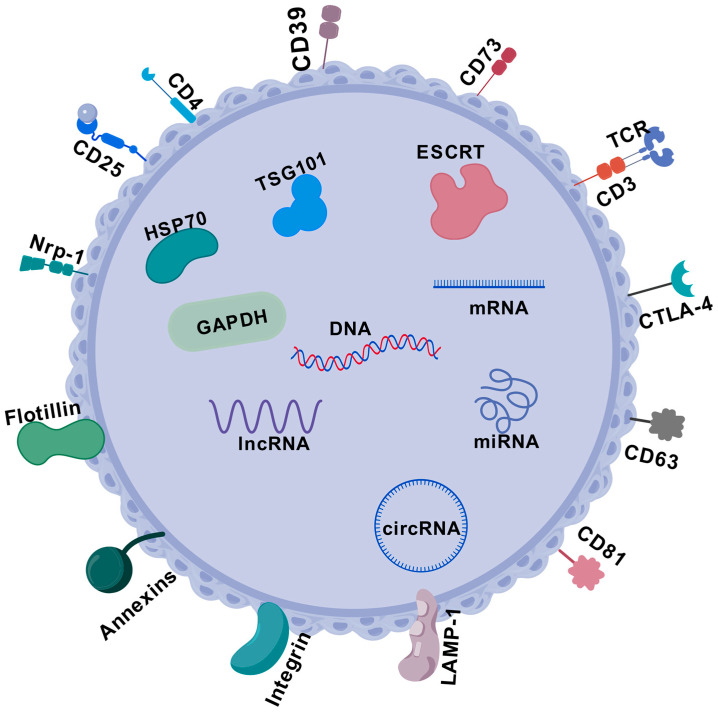
Schematic representation of the structure and composition of Treg-Evs. Treg-EV endonucleic acids: DNA, mRNA, miRNA, lncRNA and circRNA. Common proteins of Treg-EVs are classified as membrane proteins: TCR/CD3, membrane transporter proteins (flotillin), fusion proteins (annnexins), tetratransmembrane proteins (CD63), cell adhesion-associated proteins (integrins) and lysosomal-related membrane proteins (LAMP-1). Intracellular proteins: heat shock protein (HSP70), endosomal sorting complex for transport (ESCRT), auxiliary protein components (TSG101), GAPDH and cell-specific proteins related to Treg cell surface proteins (e.g., CD4, CD25, CTLA-4, CD73, CD81, etc.).

It has been demonstrated that exosomes secreted by Tregs contain a variety of non-coding RNAs (ncRNAs), microRNAs (miRNAs) and messenger RNAs (mRNAs). ncRNAs include microRNAs (miRNAs), long-chain non-coding RNAs (lncRNAs) and cyclic RNAs (circRNAs), which are widely involved in tumor signaling and phenotyping. ncRNAs are also key regulators of the immune system, regulating the development, homeostasis and function of Tregs, as well as Treg development, homeostasis and function [146,248,249,250,251,252]. After the discovery of miRNAs, it has been demonstrated that miR-142-5p post-transcriptionally inhibits the expression of the cAMP hydrolase phosphodiesterase-3b, thereby controlling the function of Tregs [253]. In BC, the lncRNA of small nucleolar RNA host gene 1 (SNHG1) competitively binds to miR-448 and inhibits the expression of IDO, thereby downregulating Treg differentiation and controlling immune escape from BC [254]. When miR-21 is silenced, the expression of the tumor suppressor phosphatase and tensor protein homologue (PTEN) goes up. This changes how the Akt pathway is activated, which in turn prevents the growth of CCR6^+^ Tregs [255]. It was found that miR-520b upregulated PTEN in MCF-7 BC cells. miR-520b inhibitor increased the expression of IFNγ and decreased the expression of Foxp3. It can 15 olarizes macrophages to M1, thus inhibiting tumor growth [256]. The detailed structure of Treg-EVs is shown in Figure 6.

### 5.10. Nano-Engineered Targeting of Tregs

More and more studies have found that systemic inhibition of Tregs is less efficient in tumor therapy, while the transcriptome of intra-tumor Tregs and peripheral Tregs has been shown to differ. Therefore, more and more scholars have started to target Tregs from nano-loaded drugs to inhibit tumor growth in order to improve the efficiency of treatment [119,257,258] (Table 1). Combining nanoparticles (NPs) with immune targeting anti-CTLA-4 antibody and anti-PD-1 antibody can increase the efficiency of immunotherapy while improving the precision [259,260]. In addition, a novel micellar nanocarrier was created by combining polyethylene glycol (PEG) with the IDO inhibitor NLG919 and incorporating a Fmoc moiety for increased stability. The carrier was packaged with paclitaxel (PTX) and delivered to a 4T1 tumor mouse model. The results showed an increase in FN-γ^+^CD4^+^ and CD8^+^ T, and a significant decrease in Tregs [261]. The combination of tLyp1 peptide-modified Tregs targeting a hybrid therapeutic NP (tLyp1-hNP) targeted Nrp1 receptors and CTLA-4 in Tregs to further enhance Teff cell function and anticancer effects [262]. Similarly, a curcumin analog (CA) encapsulated with α-lactalbumin (α-LA) constituting NPs has been used to target and inhibit the Nrp1 receptor in Tregs, resulting in anticancer effects [263]. Domvri K et al. designed specialized copper sulfide nanocarriers (CuSNC) encapsulated with Epacadostat/Dasatinib (EPDA) complexes to nano-target Tregs in a mouse model of triple-negative BC 4T1 tumors and found that CuS/EPDA inhibited metastatic BC tumor growth in combination with anti-PD-L1 drugs [264]. Other scholars combined physical anticancer therapy with nanomedicine therapy. By loading IR-780 photothermal sensitiser combined with imatinib and glucocorticoid-induced TNF receptor family-related protein (GITR-PLGA) NP, the inhibitory function of Tregs was downregulated by photothermal action and immunosuppression together to enhance the antitumor ability of Teff [265]. The use of NPs composed of highly crystalline IONPs in combination with photothermal therapy (PTT) as locally targeted therapies has also become a new research direction. It can enhance drug penetration, maintain drug concentration around tumor Tregs, inhibit the growth of 4T1 tumor mouse models, and produce a distant effect of inhibiting distal tumors [266]. Similarly, combining two different drugs together and then delivering them in vivo by nanotechnology is also a good option using a pre-drug NP (DOX/IND@NPs) based on a mouse model of BC, which delivered adriamycin (DOX) and indolimod (IND) to block the IDO pathway, further suppressing Treg levels and generating antitumor immunity [267]. Zhang L et al. found that Treg-specific YTHDF1-deficient mice inhibited melanoma tumor growth without affecting peripheral immune homeostasis, but this resulted in increased apoptosis and an impaired suppressive function of Tregs in TME [268]. Tian et al. used lonidamine (LND) and xylosepine (Sy) lactate modulators to target the interior of the TME by way of NPs, thereby increasing the number of NKs and Tregs [269]. An engineered cell membrane to PD-1 receptor-presenting membrane-encapsulated paclitaxel dimer NPs (PD-1@PTX 2 NPs) selectively binds to PD-L1 ligands on the BC surface and can release NPs, resulting in a 3.2-fold depletion of Tregs in vivo in mice, exhibiting a 71.3% tumor growth inhibition [270], using anti-CD80 antibody-modified biodegradable PCL-Hyd-PEG vesicles carrying NP (EAC-NP), combined with the HSP70 chaperoning polypeptide (HCP) and a demethylated CpG matrix (CpG ODN) adjuvant. The drug can be specifically targeted to APC and can increase the ratio of CD4^+^ T/Tregs and CD8^+^ T/Tregs, thus effectively fighting BC cells [271]. By encapsulating specific siRNAs (e.g., A2AR; CD73), NPs can be targeted against the metabolism of Tregs within the tumor to control tumor growth [237,272].

Overall, the use of genetic engineering and nanotechnology to inhibit Tregs by direct or indirect means is also a promising therapeutic approach, which exhibits a high degree of uptake and specificity that warrant a continued in-depth investigation.

### 5.11. Tregs in Combination with Radiotherapy

A growing number of studies have found that the synergistic effect of radiation therapy (RT) with immune cells enhances the antitumor effect. It also induces chemotherapeutic factors and modulates cell surface molecules to enhance T-cell recruitment [273,274,275]. Treg counts during RT can also serve as an important indicator of dose adjustment [276]. Not only can RT achieve therapeutic effects in terms of systemic immune cells through Treg, but it was also found that in a poorly immunogenic mouse cancer model, RT treatment after the blockade of TGFβ effectively generated CD8^+^ T cell responses against multiple endogenous tumor antigens and inhibited tumor growth [277]. Further studies found that RT combined with a TGFβ inhibitor effectively inhibited Treg infiltration in mice with low levels of activin A (TSA) BC, but showed the opposite result in a 4T1 BC tumor model with high activin A expression [196]. To enhance the distant effect (AE) of RT, recent studies have shown that the combination of the anti-alcohol drug disulfiram (DSF) with copper (DSF/Cu) complexes and RT can reduce the number of Tregs, and increase the number of CD8^+^ and CD4^+^ cells in a 4T1 BC mouse model [278]. Such studies provide new insights into the Treg pathway for antitumor therapy and contribute to the development of new therapeutic strategies for tumor immunosuppression.

## 6. Challenges

Although the results of preclinical studies are encouraging, challenges remain in translating Treg-targeted therapies into clinical practice. Achieving specificity in targeting Tregs without compromising systemic immune tolerance is a major hurdle. Tregs maintain immune homeostasis in the immune system. The systemic depletion of Tregs may have adverse consequences. The main challenge is its potential to trigger serious immune-related adverse reactions (irAEs) [279,280], and inflammatory damage to organs such as colitis and dermatitis, and rare diseases such as aplastic anemia [281,282,283], since Tregs exhibit a high degree of heterogeneity. In the TME, they are predominantly immunosuppressive, whereas in the peripheral blood, they mainly maintain immune homeostasis. Therefore, the specific depletion of Tregs is a challenging task [284]. In addition, many of Treg targets (e.g., CTLA-4, PD-1, GITR, OX40, etc.) are expressed in other types of immune cells, and in particular, Teff and antigen-presenting cells may attenuate the specificity of treatment [285,286]. There are other factors in the TME (e.g., TGF-β, VEGF, etc.) that promote the recruitment and amplification of Tregs. Once targeted, Tregs may trigger other immune mechanisms to promote compensation [287]. All these reasons may complicate the treatment of targeted Tregs. Current research suggests that anti-CTLA4 has more side effects than anti-PD-1, possibly because anti-PD-1 targets the TME, while anti-CTLA4 has a more aggregated effect [288,289]. In preclinical trials, the probability of irAEs was found to be 90% and 70% for anti-CTLA4 and anti-PD-1, respectively [290]. Ipilimumab alone does cause significant adverse effects based on multi-organ inflammation. More severe irAEs occur when combination therapy is used [291]. In order to improve targeting ability and reduce side effects, it has been proposed that the transient depletion of Tregs, short-term depletion, can inhibit the growth of 4T1.2 BCs and reduce the occurrence of irAEs [292]. Dees et al. proposed the selective depletion of tumor-infiltrating Tregs while retaining less inhibitory naïve and peripheral Tregs to reduce the incidence of irAE [293]. Some scholars also found that tumor-specific co-suppressor receptors (e.g., TIM3, LAG3, and TIGIT, etc.) are highly expressed in a subpopulation of tumor-infiltrating Tregs. Thus, tumor-specific co-suppressor receptors started to be targeted in combination with photoimmunotherapy and the induction of local Tregs depletion [289,294]. In addition, Treg metabolic pathway inhibitors (e.g., TLR8) and nano-engineered Tregs can also contribute to improved targeting selectivity [295,296].

## 7. Future Directions

In the future, it is possible to combine Tregs as carriers with nano-anticancer drugs. Tregs play an immunomodulatory role in the TME, whereas anticancer drugs act directly on tumor cells. This approach can highlight the advantages of Tregs in the TME. It also improves the anticancer effect and reduces systemic toxicity. The relationship between Treg-EV and ASC-EV in BC can also be further investigated, along with the development of a novel biological nanocarrier system via special exosomes. Future nano-engineered Tregs represent an emerging and promising therapeutic strategy. Targeting tumors or other pathological states more effectively is possible by combining the precise delivery of nanotechnology with the immunomodulatory function of Tregs. Although most of the studies are still in the preclinical stage, the rapid development of this field holds promise for the future treatment of cancer immunotherapy and other immune-related diseases.

## 8. Conclusions

Tregs play a complex role in BC progression, exerting immunosuppressive and regulatory functions in TME. Although the presence of Tregs is associated with a poor prognosis, recent advances in immunotherapy targeting Tregs provide new avenues for interventions. By selectively modulating Treg activity and quantity, the maximum potential of the immune system against BC is unleashed, while minimizing off-target effects. Ongoing research aimed at unravelling the complexity of Treg biology and optimizing therapeutic strategies is expected to improve outcomes for BC patients.

## Figures and Tables

**Table 1:** NP targeting of Treg-specific cells.

Nanocarrier	Size (nm)	Supportive Measures	Treg Level	Other Marker Levels	Breast Cancer Model	Ref.
Lipid	176.5 ± 62.23 nm	UA	Down	pSTAT5; IL-10; IL-6 ↓	4T1 mice	[267]
PEG2k-Fmoc-NLG	90 nm	PTX	Down	IFN-γ^+^ CD4^+^; CD8^+^ T; M1 Macrophages; G-MDSC ↑ M2 Macrophages ↓	4T1.2 mice	[259]
DOX/IND@NPs	104 ± 3.21 nm	IND+DOX	Down	CD8^+^ T ↑; VEGF; MMP9; CD31 ↓	4T1 mice	[259]
PNT/DOX NPs	232.0 ± 11.5 nm	DOX+R837	Down	IL-6; IL-12; TNF-α; IFN-γ; DC ↑	4T1 mice	[267]
L@S/L	100 nm	LND+Sy	Down	M1 Macrophages; NK ↑ M2 Macrophages; Lactic acid ↓	4T1 mice	[267]
PEG2k-Fmoc-IL36	<450 nm	Anti-CTLA-4mAb	Down	IFN-γ^+^ CD4^+^T ↑ IFN-γ^+^ CD8^+^ T ↑	4T1.2 Lung metastasis mice	[259]
ChLa NPs	100 nm	CD73-specific siRNA+DC vaccine	Down	IFN-γ ↑ CD73; A2AR; IL-10; IL-17 ↓	4T1 mice	[259]
PCL NPs	100 nm	A2AR-specific siRNA	Down	IFN-γ ↑ IL-10; A2AR ↓	4T1 mice	[259]
PD-1@PTX 2 NPs	203.7 nm	PTX	Down	TNF-α; IFN-γ ↑; CD8^+^ T ↑ (3.2 times)	4T1 mice	[259]
DACss	73.67 ± 1.80 nm	DMC+Anti-PD-1mAb+PDT	Down	ROS ↑ CD80^+^ CD86^+^ DC ↑	4T1 mice	[270]
CA@α-LA	175.0 ± 8.4 nm	Anti-Nrp-1 mAb	Down	Caspase-3; Bcl-2 ↑ VEGF ↓	4T1 mice	[259]
Polymer-coated IONPs	30.7 ± 1.8 nm	Anti-CTLA-4 mAb+PTT	Down	CD8^+^ T ↑	4T1 mice	[270]
CuS	84 ± 3.0 nm	EPDA +PTT	Down	ROS ↑ Ki-67; IDO-1; c-KIT; EPHA2; PDGFRβ ↓	4T1 mice	[259]
PCL-Hyd-PEG	150 nm	Tumor endogenous antigens (HCP)+ adjuvants CpG ODN+ Anti-CD80 mAb	Down	IFN-γ; CD4^+^ T; CD8^+^ T ↑	4T1 rat	[259]

Abbreviations: UA—Ursolic acid; pSTAT5—transcription 5; PEG—polyethylene glycol; PTX—paclitaxel; G-MDSC—granulocytic myeloid-derived suppressor cell; IND—indoximod; DOX—doxorubicin; NPs—nanoparticles; MMP—matrix metalloproteinase; R837—imiquimod; LND—lonidamine; Sy—syrosingopine; ChLa—chitosan–lactate; PCL—PEG–chitosan–lactate; DMC—demethylcantharidin; PDT/PTT—photodynamic therapy; DACss—supramolecular photodynamic nanoparticles; OS—reaction oxygen; CA—curcumin analog; α-LA—α-lactalbumin; Bcl-2—B cell lymphoma-2; IONPs—iron-oxide nanoparticles; CuS—copper sulfide; EPDA—epacadostat/dasatinib; PCL—poly ε-caprolactone.
